# False start: Non-compliance with Victoria’s new Mental Health and Wellbeing Act

**DOI:** 10.1177/10398562251383760

**Published:** 2025-11-02

**Authors:** Simon Katterl

**Affiliations:** Simon Katterl Consulting, Melbourne, Australia

**Keywords:** human rights, clinical governance, leadership, mental health law

## Abstract

**Objective:**

To assess the compliance of Victorian designated mental health services (DMHS) with mental health and wellbeing principles under section 30 of the *Mental Health and Wellbeing Act 2022* (Vic).

**Method:**

An audit of 19 Victorian DMHS’ annual reports to assess compliance with a duty to report actions on one or more mental health and wellbeing principles (principles) within the last year.

**Results:**

16 of 19 (84.2%) of designated mental health services breached their duty to report on how they complied with one or more principles within the last year. The three compliant services provided varied responses to this duty.

**Conclusions:**

The vast majority of Victorian DMHS have failed to comply with a minimum reporting requirement under the MHWA. This data suggests that imprecise legislative drafting, an inadequate commissioning and regulatory framework as well as under-enforcement by the Mental Health and Wellbeing Commission may have contributed to non-compliance. Further research may examine how regulatory oversight agencies can ensure greater compliance with human rights and the MHWA.

Mental health laws often undermine human rights; their arbitrary application erodes the rule of law. This is true in several senses. Mental health laws that permit the use of substitute decision-making regimes and coercive practices are assessed by some authoritative bodies to be inconsistent with the Convention on the Rights of Persons with Disabilities.^1^ Even within the operation of mental health laws, such as in Victoria, Australia, there are significant divergences in the use of compulsory treatment and seclusion and restraint between designated mental health services (DMHS).^2^ Implementation of principles detailed within Victorian mental health laws was at best, a patchwork.^2^ This divergence, as well as research^3,4^ and inquiry findings^2^ reinforce the conclusion that Victoria’s public mental health system often utilises the law to use compulsory treatment, seclusion, and restraint, but fails to comply with countervailing duties that regulate and minimise their use. As such, regulatory oversight processes that give effect to such laws have been questioned.^5^ The system, it may appear, uses the law, but is simultaneously above it.

The Royal Commission into Victoria’s Mental Health System (Royal Commission) made recommendations to change this. Arising from the 74 recommendations, the *Mental Health and Wellbeing Act 2022* (Vic) (MHWA) replaced the *Mental Health Act 2014* (Vic) (the previous Act) on 1 September 2023. The Victorian Parliament, through the MHWA, has defined the contemporary legal basis for DMHS to operate and what conduct is permissible. While rhetorically shifting towards a more rights-based mental health system, the MHWA only made relatively minor changes in terms of human rights protections for people accessing public mental health services. Instead, much focus of the legislation was to establish key statutory bodies, including the Mental Health and Wellbeing Commission (MHWC), the Victorian Collaborative Centre for Mental Health and Wellbeing and Youth Mental Health Victoria.

One of the substantive rights-related changes to the MHWA was the introduction of new mental health and wellbeing principles (sections 16 to 28, principles). Such principles, sitting alongside treatment decision-making principles (sections 79 to 83), were intended to guide both clinical practice and the governance, design, and operation of DMHS. Sections 16 to 28 of the MHWA outline 13 principles detailed in Table 1 below. Under the MHWA, DMHS have different compliance and reporting duties. Subsections 29(a) requiring all DMHS take all reasonable efforts to comply with the principles and subsection 29(b) requiring that DMHS give proper consideration to the principles.^6^

**Table 1.** Mental health principles under the *Mental Health and Wellbeing Act 2022* (vic)

• Dignity and autonomy principle
• Diversity of care principle
• Least restrictive principle
• Supported decision making principle
• Families and carers principle
• Lived experience principle
• Health needs principle
• Dignity of risk principle
• Wellbeing of young people principle
• Diversity principle
• Gender safety principle
• Cultural safety principle
• Wellbeing of dependents principle

Section 30 creates a reporting obligation on DMHS by requiring that they must include in their annual report ‘information about *actions taken* during the reporting period that *relate to giving effect to one or more of the mental health and wellbeing principles*’ (emphasis added). No supportive commentary is found in the Parliamentary second reading speech or from Victorian Government publications. Interpreting this provision based on a construction that promotes the purpose and objects of the MHWA (the test required under section 35(a) of the *Interpretation of Legislation Act 1984* (Vic)), and operates similarly to section 29(b) makes clear that the requirement necessitates DMHS make reports that demonstrate an explicit connection between one or mental health principles and actions taken. Merely reporting general actions with no nexus to relevant principles, as may have been done in previous years, would not reflect the intention of Parliament in introducing this provision. The first year of annual reporting following the introduction of the MHWA was the 2023–2024 financial year.

This paper audits the compliance of 19 DMHS against their duties under section 30 of the MHWA. The audit aims to identify which of the 19 DMHS documented their actions to implement one or more of the principles under the MHWA in their annual report. This audit provides an insight into whether DMHS are complying with their legal obligations under the MHWA.

## Method

19 annual reports were retrieved for the 2023–2024 period for the DMHS outlined under Table 2. These annual reports were retrieved from the Parliament of Victoria tabled documents database. Each designated mental health service’s annual report was assessed for reporting actions on one or more of the 13 principles of the MHWA, with the text from the annual reports captured. No ethics approval was required as this data is drawn from publicly available sources that does not identify individuals.

**Table 2.** Victorian DMHS

• Albury Wodonga Health (7)
• Alfred Health (8)
• Austin Health (9)
• Barwon Health (10)
• Bendigo Health (11)
• Eastern Health (12)
• Goulburn Valley Health (13)
• Grampians Health (14)
• Latrobe Regional Hospital (15)
• Melbourne Health (16)
• Mercy Public Hospitals Incorporated (17)
• Mildura Base Public Hospital (18)
• Monash Health (19)
• Northern Health (20)
• Peninsula Health (21)
• SouthWest Healthcare (22)
• St Vincent’s Hospital (Melbourne) Limited (23)
• The Royal Children’s Hospital Melbourne (24)
• Western Health (25)

## Results

16 of 19 DMHS failed to comply with section 30 of the MHWA which requires public reporting on actions taken to comply with one or more of the principles (see Table 3). Three services – Eastern Health, Peninsula Health, and Bendigo Health – were the only services that complied with this duty. This reflects a breach of section 30 of the MHWA by 84.2% of Victorian DMHS.

**Table 3.** Results of audit

Service	Complied/Breached
Albury Wodonga Health	Breached
Alfred Health	Breached
Austin Health	Breached
Barwon Health	Breached
Bendigo Health Care Group	Complied
Eastern Health	Complied
Goulburn Valley Health	Breached
Grampians Health	Breached
Latrobe Regional Hospital	Breached
Melbourne Health	Breached
Mercy Public Hospitals Incorporated	Breached
Mildura Base Public Hospital	Breached
Monash Health	Breached
Northern Health	Breached
Peninsula Health	Complied
South West Healthcare	Breached
St Vincent’s Hospital (Melbourne) Limited	Breached
The Royal Children’s Hospital	Breached
Western Health	Breached

Each of these three DMHS reported their compliance in differing ways. Components of the three service responses in annual reports are included in Table 4. The length varied between Peninsula Health (105 words), Eastern Health (187 words) and Bendigo Health (368 words). Whereas Peninsula Health reported on work it appeared to have already been undertaking, Bendigo Health and Eastern Health reported on specific actions and roles that arose from consideration of specific MHWA principles. Some services reported on actions that could be inferred as relating to principles, such as matters in relation to seclusion and restraint, but no connection was made to principles in that reporting.

**Table 4.** Reporting from compliant DMHS

DMHS	Principle reported on	Response
Bendigo Health	Dignity and autonomy (section 16), diversity of care (section 17) gender safety (section 26)	‘Through the implementation of the Mental Health and Wellbeing Act 2022 (MHWA22) Bendigo Health’s Mental Health and Wellbeing (MHW) directorate reviewed all training, staff and consumer resources, advocacy programs, feedback pathways and policies and protocols. This was to ensure the principles of the act, including dignity and autonomy, was accurately reflected and implemented in a meaningful and sustainable way’.
		Bendigo Health makes consumers and their support persons aware of their rights and responsibilities and how they can exercise them while receiving treatment and support – either voluntarily or under the MHWA22 – as part of standard care provision. Information is provided verbally and in written communication. It is reiterated throughout a person’s care, in a language and manner that best suits their needs. Information regarding independent advocacy agencies is provided by the service and staff to help consumers get further support to exercise their rights. Multiple options for feedback from consumers are in place and continually reviewed by Bendigo Health.… specific service roles have been created to further strengthen work relevant to the principles of Diversity and Gender. The roles include; a mental health spiritual care practitioner, Koorie Mental Health Liaison Officer (both inpatient and community based), Women’s mental health workers and mental health peer support workers. Work to strengthen external partnerships continues. This includes; formal partnerships with MIND Australia, the Bendigo and District Aboriginal Co-operative (BDAC), Njernda Aboriginal Corporation and the Mallee District Aboriginal Services (MDAS). Partnerships have been created with Thorne Harbour Health and local alcohol and other drug services in the delivery of care and the establishment of Local Mental Health and Wellbeing services in Bendigo and Echuca. Refurbishment works to improve the physical environment of the Intensive Care Area (ICA) of the Adult Acute inpatient Unit (AAU) will start in coming months.’ (11)
Eastern Health	Dignity and autonomy principle (section 16)	‘Eastern Health’s mental health and wellbeing program acknowledges the mental health and wellbeing principles as stated in the Mental Health and Wellbeing Act 2022 (Vic).
		Eastern health aims to promote conditions in which people can experience good mental health and wellbeing, aiming to provide treatment and care in the community that is compassionate, safe and of a high quality, as close to home as possible. The mental health and wellbeing program, in responding specifically to the principles, acknowledges the principle of dignity and autonomy and respects the rights of people living with mental illness or psychological distress, recognising that the person is to be supported to exercise their rights. The program maintains close working relationships with peak advocacy bodies in ensuring consumers can express their rights. The program has recently commenced a quality project across both bed-based and community services to increase the number of consumers who have a documented advance statement of preferences available in their clinical record. Training has been developed to assist clinicians and medical personnel to improve their understanding of and capacity to support consumers to complete an advance statement of preferences.’ (12)
Peninsula Health	Dignity and autonomy (section 16)	‘Peninsula Health is a leader in the reduction of restrictive interventions in Australia. The mental health and wellbeing service team has continued its sector-leading work in this area, and has eliminated the use of seclusion, while maintaining low rates of physical restraints and maintaining staff safety. Peninsula Health is currently engaged in multiple research projects to further evaluate the nature of systems change and culture underlying this success, while supporting the development of further improvements. The mental health and wellbeing service regularly hosts learning visits from other services across Victoria and the rest of Australia, to provide teaching in how to implement these successes elsewhere’ (21)
	Least restrictive (section 18)	

## Discussion

This audit has found that the overwhelming majority of Victorian DMHS breached the MHWA less than 1 year after its commencement. The audit revealed that more than four-fifths of services did not report in their annual report on the actions they took to comply with one or more of the MHWA principles, as required under section 30. The audit also revealed significant differences between the three DMHS in how they reported on their compliance with the principles. Of those that complied, some appeared to report that the MHWA principles prompted changes to take place within the service, while another appeared to state that principles merely reflected what was already underway. That the regulatory burden of complying with section 30 is so modest, and yet so widely breached, presents a bleak picture regarding DMHS’ compliance with the MHWA.

The evidence from this audit is consistent with previous research illustrating breaches of Victoria’s mental health laws. The Royal Commission found that the previous mental health principles had not been adequately implemented and that authorised psychiatrists may not be considering their legal obligations before administering treatment.^2^ A subsequent study found that breaches of informed consent and other rights under the previous Act were so common as to the rights of consumers in the study ‘illusory’.^4^ This evidence is consistent with regulatory scholarship on ‘motivational postures’ that speak to the fact that DMHS will have varying degrees of support for legislative requirements under the MHWA.^3^ These findings contribute to a body of research that indicates DMHS that utilise the law to deliver compulsory treatment are often operating outside of its bounds.

There may be several reasons for non-compliance with these duties. Resourcing may be identified as an issue. However, there has been a significant increase in funding to the Victorian mental health system since the Royal Commission, amounting to over $6 billion into the Victorian mental health system since 2018.^26^ Much funding focused capital and other infrastructure investments into DMHS^26^ as well as ‘Act Implementation Leads’ in every DMHS.^27^ While the effect of section 30 isn’t undermined, the drafting of the provision could have been clearer and may have confused DMHS leaders about their duties. The lack of guidance from the Victorian Government on the duty may have compounded this uncertainty. A lack of accountability by DMHS was raised during the Royal Commission and may be continuing post-inquiry.^2^ Previous scholarship points to a series of regulatory failures within the Mental Health Complaints Commission (MHCC), including chronic under-enforcement.^5^ Subsequent inquiries, including the Yoorrook Justice Commission, reported on evidence ‘that the MHWC is a rebrand of the previous Mental Health Complaints Commission’ that had ‘drastically failed to provide effective oversight’.^28^

Limitations of this audit must be acknowledged. The reporting obligation under section 30 of the MHWA is one measure of compliance with the MHWA, so should be read alongside other evidence regarding compliance with Victoria’s mental health laws.^2,4^ Another limitation refers to the lack of standard-setting within section 30 duty itself: the opportunity to select a single principle and provide an account of compliance without the requirement for this to be evidenced or applied across all principles provides an incomplete and potentially misleading account of quality and compliance within DMHS.

The findings have policy and regulatory implications. Continued questions must be asked about the Victorian Government’s stewardship of the mental health system towards a system that meets human rights standards or, at a bare minimum, meets existing legislative standards under the MHWA. As part of these inquiries, it should be asked why more rigorous proposals for reporting, transformation and culture change under the MHWA were not introduced to Parliament.^29^ Yoorrook Justice Commission recommendations to address the failure to implement cultural safety principles for Aboriginal Victorians focused on an enhanced regulatory framework and better annual reporting from services.^28^ Similar questions should be asked about service commissioning processes in light of the Victorian Government withdrawing its commitment to establish Regional Mental Health and Wellbeing Boards^30^ that were tasked with co-commissioning mental health services. How this trend of non-compliance continues while the MHWC continues to under-enforce legal obligations is a question whose answer grows more urgent.

The findings encourage future research. Future research may explore why there are such divergent levels of compliance with legislative obligations. The directions, resourcing and capability support provided by the Victorian Government to DMHS may be examined, including whether appropriate, or indeed any guidance was provided for how DMHS must meet the provision’s intent. Similarly, the exploration of the interactions, recommendations and enforcement actions between the Chief Psychiatrist, the MHWC and the DMHS they regulate may explain why such non-compliance and variance continue. Finally, empirical studies that build a picture on the motivational posture of DMHS and their formal leaders may illustrate bottom-up drivers of rights breaches and rights-leading cultures and practices.^3^

## Conclusion

Victoria’s public mental health system continues to operate outside of the permissible grounds set out by Parliament. Audits of routine reporting requirements under section 30 of the MHWA reveal that the vast majority of DMHS were in breach. Immediate actions may come in the form of guidance from the Victorian Government in subordinate regulations or from the MHWC. Genuine improvements in human rights following Victoria’s Royal Commission will require a combination of legislative reform, greater capability support and improved service-level transparency. Absent improved regulatory oversight from the MHWC, agencies such as the Victorian Auditor General’s Office or the Victorian Ombudsman could undertake further review.
